# Methyl 11,14,16-triphenyl-8,12-dioxa-14,15-di­aza­tetra­cyclo­[8.7.0.0^2,7^.0^13,17^]hepta­deca-2(7),3,5,13(17),15-penta­ene-10-carboxyl­ate

**DOI:** 10.1107/S1600536813015213

**Published:** 2013-06-08

**Authors:** J. Kanchanadevi, G. Anbalagan, D. Kannan, B. Gunasekaran, V. Manivannan, N. Bakthadoss

**Affiliations:** a Department of Physics, Velammal Institute of Technology, Panchetty, Chennai 601 204, India; b Department of Physics, Presidency College (Autonomous), Chennai 600 005, India; c Department of Organic Chemistry, University of Madras, Guindy Campus, Chennai 600 025, India; d Department of Physics & Nano Technology, SRM University, SRM Nagar, Kattankulathur, Kancheepuram Dist, Chennai 603 203, Tamil Nadu, India; e Department of Research and Development, PRIST University, Vallam, Thanjavur 613 403, Tamil Nadu, India; f Department of Organic Chemistry, University of Madras, Maraimalai Campus, Chennai 600 025, India

## Abstract

In the title compound, C_33_H_26_N_2_O_4_, the pyrazole ring makes dihedral angles of 15.13 (7) and 60.80 (7)° with the adjacent phenyl rings. Both di­hydro­pyran rings exhibit half-chair conformations. A weak intra­molecular C—H⋯O inter­action occurs. In the crystal, mol­ecules are linked into inversion dimers through pairs of C—H⋯N inter­actions. Weak C—H⋯π inter­actions are also observed.

## Related literature

For the biological activity of 4*H*-chromenes, see: Cai *et al.* (2006 ▶); Gabor (1988 ▶); Brooks (1998 ▶); Valenti *et al.* (1993 ▶); Tang *et al.* (2007 ▶). For a related structure, see: Ponnusamy *et al.* (2013 ▶).
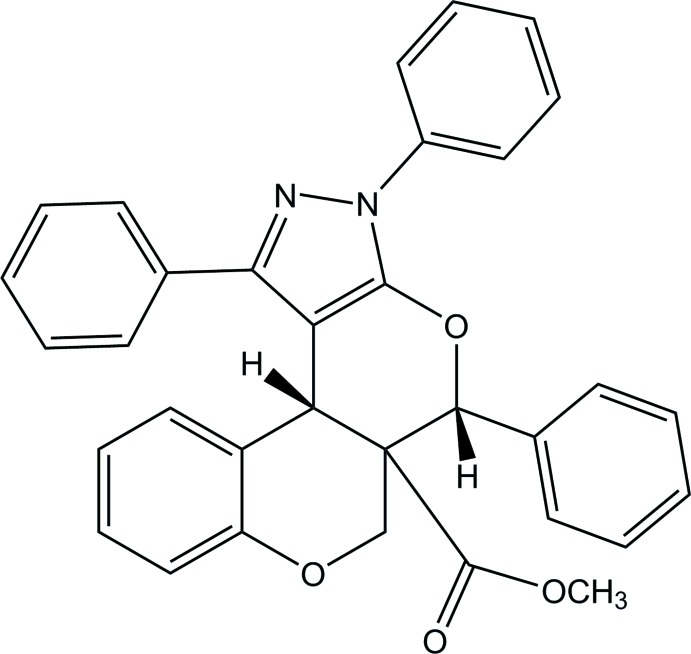


## Experimental

### 

#### Crystal data

C_33_H_26_N_2_O_4_
*M* *_r_* = 514.56
Monoclinic, 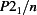

*a* = 11.916 (5) Å
*b* = 10.876 (5) Å
*c* = 21.153 (5) Å
β = 105.797 (5)°
*V* = 2637.9 (18) Å^3^
*Z* = 4
Mo *K*α radiation
μ = 0.09 mm^−1^
*T* = 295 K
0.30 × 0.20 × 0.20 mm

#### Data collection

Bruker APEXII CCD diffractometer
Absorption correction: multi-scan (*SADABS*; Sheldrick, 1996 ▶) *T*_min_ = 0.980, *T*_max_ = 0.983
40448 measured reflections
4605 independent reflections
3289 reflections with *I* > 2σ(*I*)
*R*_int_ = 0.050

#### Refinement

*R*[*F* ^2^ > 2σ(*F* ^2^)] = 0.040
*wR*(*F* ^2^) = 0.129
*S* = 1.01
4605 reflections
354 parameters
H-atom parameters constrained
Δρ_max_ = 0.22 e Å^−3^
Δρ_min_ = −0.15 e Å^−3^

### 

Data collection: *APEX2* (Bruker, 2008 ▶); cell refinement: *SAINT* (Bruker, 2008 ▶); data reduction: *SAINT*; program(s) used to solve structure: *SHELXS97* (Sheldrick, 2008 ▶); program(s) used to refine structure: *SHELXL97* (Sheldrick, 2008 ▶); molecular graphics: *PLATON* (Spek, 2009 ▶); software used to prepare material for publication: *SHELXL97*.

## Supplementary Material

Crystal structure: contains datablock(s) I. DOI: 10.1107/S1600536813015213/is5278sup1.cif

Structure factors: contains datablock(s) I. DOI: 10.1107/S1600536813015213/is5278Isup2.hkl

Supplementary material file. DOI: 10.1107/S1600536813015213/is5278Isup3.cml

Additional supplementary materials:  crystallographic information; 3D view; checkCIF report

## Figures and Tables

**Table 1:** Hydrogen-bond geometry (Å, °) *Cg*1, *Cg*4 and *Cg*6 are the centroids of the N1/N2/C7/C24/C25, C1–C6 and C17–C22 rings, respectively.

*D*—H⋯*A*	*D*—H	H⋯*A*	*D*⋯*A*	*D*—H⋯*A*
C18—H18⋯N2^i^	0.93	2.62	3.517 (3)	163
C6—H6⋯O1	0.93	2.26	2.877 (2)	123
C13—H13⋯*Cg*6^ii^	0.93	2.98	3.904 (8)	174
C18—H18⋯*Cg*1^i^	0.93	2.88	3.720 (5)	150
C23—H23⋯*Cg*4^iii^	0.98	2.86	3.787 (6)	159

Symmetry codes: (i) 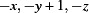; (ii) 
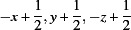
; (iii) 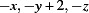.

**Fig. 1.**
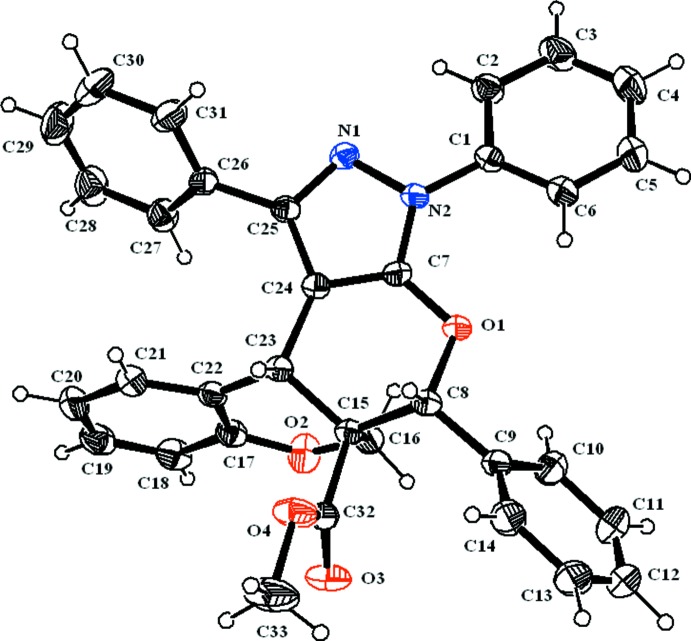
The molecular structure of the title compound, with atom labels and 30% probability displacement ellipsoids for non-H atoms.

**Fig. 2.**
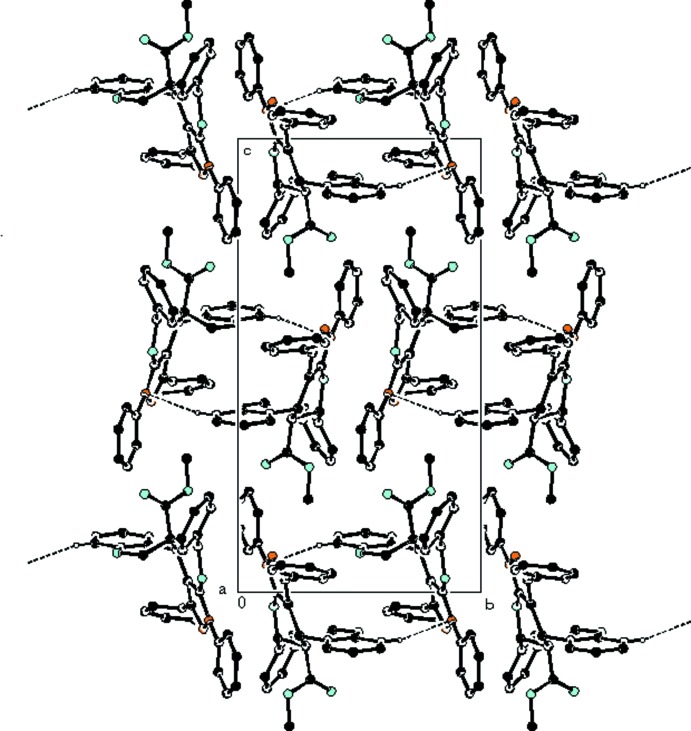
A packing diagram of the title compound, viewed down the a axis. Hydrogen bonds are shown as dashed lines.

## supplementary crystallographic information

### Comment

4*H*-Chromenes and their derivatives exhibit various biological activities such as anti-viral, anti-fungal, anti-inflammatory, antidiabetic, anti-anaphylactic and anti-cancer (Cai *et al.*, 2006; Gabor, 1988; Brooks, 1998; Valenti *et al.*, 1993; Tang *et al.*, 2007).

The geometric parameters of the title molecule (Fig. 1) agree well with a reported similar structure (Ponnusamy *et al.*, 2013). The pyrazole ring makes dihedral angles of 15.13 (7) and 60.80 (7)°, respectively, with two phenyl (C1–C6) and (C26–C31) rings. The molecular structure is stabilized by a weak intramolecular C—H···O interaction and the crystal packing is controlled by weak intermolecular C—H···N and C—H···π interactions (Table 1).

### Experimental

A mixture of (*E*)-methyl-2-[(2-formylphenoxy)methyl]-3-phenylacrylate (0.296 g, 1 mmol) and 1,3-diphenyl-4,5-dihydro-1*H*-pyrazol-5-one (0.236 g, 1 mmol) was placed in a round bottom flask and melted at 180 °C for 1 h. After completion of the reaction as indicated by TLC, the crude product was washed with 5 ml of ethylacetate and hexane mixture (1:49 ratio) which successfully provided the title compound as colorless solid in 93% yield.

### Refinement

H atoms were positioned geometrically and refined using riding model, with C—H = 0.93 Å and *U*_iso_(H) = 1.2*U*_eq_(C) for aromatic C—H, C—H = 0.98 Å and *U*_iso_(H) = 1.2*U*_eq_(C) for methine, C—H = 0.97 Å and *U*_iso_(H) = 1.2*U*_eq_(C) for methylene, and C—H = 0.96 Å and *U*_iso_(H) = 1.5*U*_eq_(C) for methyl.

### Crystal data

**Table:**

C_33_H_26_N_2_O_4_	*F*(000) = 1080
*M**_r_* = 514.56	*D*_x_ = 1.296 Mg m^−^^3^
Monoclinic, *P*2_1_/*n*	Mo *K*α radiation, λ = 0.71073 Å
Hall symbol: -P 2yn	Cell parameters from 9160 reflections
*a* = 11.916 (5) Å	θ = 2.3–25.1°
*b* = 10.876 (5) Å	µ = 0.09 mm^−^^1^
*c* = 21.153 (5) Å	*T* = 295 K
β = 105.797 (5)°	Block, colourless
*V* = 2637.9 (18) Å^3^	0.30 × 0.20 × 0.20 mm
*Z* = 4	

### Data collection

**Table:**

Bruker APEXII CCD diffractometer	4605 independent reflections
Radiation source: fine-focus sealed tube	3289 reflections with *I* > 2σ(*I*)
Graphite monochromator	*R*_int_ = 0.050
Detector resolution: 0 pixels mm^-1^	θ_max_ = 25.1°, θ_min_ = 2.3°
ω and φ scans	*h* = −14→14
Absorption correction: multi-scan (*SADABS*; Sheldrick, 1996)	*k* = −12→12
*T*_min_ = 0.980, *T*_max_ = 0.983	*l* = −24→24
40448 measured reflections	

### Refinement

**Table:**

Refinement on *F*^2^	Secondary atom site location: difference Fourier map
Least-squares matrix: full	Hydrogen site location: inferred from neighbouring sites
*R*[*F*^2^ > 2σ(*F*^2^)] = 0.040	H-atom parameters constrained
*wR*(*F*^2^) = 0.129	*w* = 1/[σ^2^(*F*_o_^2^) + (0.0765*P*)^2^ + 0.2117*P*] where *P* = (*F*_o_^2^ + 2*F*_c_^2^)/3
*S* = 1.01	(Δ/σ)_max_ < 0.001
4605 reflections	Δρ_max_ = 0.22 e Å^−^^3^
354 parameters	Δρ_min_ = −0.15 e Å^−^^3^
0 restraints	Extinction correction: *SHELXL*, Fc^*^=kFc[1+0.001xFc^2^λ^3^/sin(2θ)]^-1/4^
Primary atom site location: structure-invariant direct methods	Extinction coefficient: 0.0055 (11)

### Fractional atomic coordinates and isotropic or equivalent isotropic displacement parameters (Å^2^)

**Table:**

	*x*	*y*	*z*	*U*_iso_*/*U*_eq_	
C1	0.05478 (14)	0.91979 (14)	−0.10983 (8)	0.0365 (4)	
C2	−0.00894 (17)	0.91873 (17)	−0.17503 (8)	0.0485 (5)	
H2	−0.0865	0.8936	−0.1868	0.058*	
C3	0.0436 (2)	0.9554 (2)	−0.22240 (9)	0.0640 (6)	
H3	0.0008	0.9555	−0.2663	0.077*	
C4	0.1577 (2)	0.9914 (2)	−0.20573 (11)	0.0709 (6)	
H4	0.1929	1.0147	−0.2381	0.085*	
C5	0.21952 (19)	0.9930 (2)	−0.14101 (11)	0.0678 (6)	
H5	0.2970	1.0184	−0.1295	0.081*	
C6	0.16912 (16)	0.95781 (18)	−0.09264 (9)	0.0525 (5)	
H6	0.2119	0.9597	−0.0487	0.063*	
C7	0.04554 (14)	0.84233 (14)	0.00130 (8)	0.0340 (4)	
C8	0.18737 (14)	0.83563 (15)	0.10115 (8)	0.0365 (4)	
H8	0.1595	0.9084	0.1196	0.044*	
C9	0.31735 (15)	0.83154 (16)	0.12662 (8)	0.0414 (4)	
C10	0.38724 (16)	0.77182 (19)	0.09357 (10)	0.0559 (5)	
H10	0.3532	0.7349	0.0532	0.067*	
C11	0.50601 (18)	0.7664 (2)	0.11951 (13)	0.0738 (7)	
H11	0.5519	0.7268	0.0966	0.089*	
C12	0.5564 (2)	0.8188 (3)	0.17848 (14)	0.0864 (9)	
H12	0.6365	0.8122	0.1968	0.104*	
C13	0.4900 (2)	0.8812 (3)	0.21107 (12)	0.0845 (8)	
H13	0.5255	0.9191	0.2509	0.101*	
C14	0.37027 (18)	0.8886 (2)	0.18530 (10)	0.0611 (6)	
H14	0.3256	0.9320	0.2076	0.073*	
C15	0.12185 (14)	0.72167 (14)	0.11748 (7)	0.0342 (4)	
O2	0.08181 (11)	0.50306 (10)	0.08741 (7)	0.0573 (4)	
C17	−0.02322 (16)	0.51735 (15)	0.09970 (8)	0.0433 (4)	
C18	−0.07915 (18)	0.40980 (18)	0.10863 (9)	0.0550 (5)	
H18	−0.0453	0.3342	0.1045	0.066*	
C19	−0.18357 (19)	0.4139 (2)	0.12349 (10)	0.0625 (6)	
H19	−0.2216	0.3412	0.1283	0.075*	
C20	−0.23285 (18)	0.5254 (2)	0.13141 (10)	0.0618 (6)	
H20	−0.3031	0.5285	0.1426	0.074*	
C21	−0.17688 (16)	0.63237 (18)	0.12258 (9)	0.0518 (5)	
H21	−0.2099	0.7076	0.1283	0.062*	
C22	−0.07280 (14)	0.63042 (15)	0.10545 (8)	0.0381 (4)	
C23	−0.01121 (14)	0.74599 (14)	0.09516 (8)	0.0340 (4)	
H23	−0.0295	0.8107	0.1230	0.041*	
C24	−0.03910 (14)	0.79328 (14)	0.02535 (8)	0.0332 (4)	
C25	−0.14127 (14)	0.80602 (14)	−0.02772 (8)	0.0354 (4)	
C26	−0.26381 (14)	0.77063 (16)	−0.03464 (8)	0.0393 (4)	
C27	−0.29485 (17)	0.64951 (17)	−0.03038 (9)	0.0502 (5)	
H27	−0.2372	0.5894	−0.0199	0.060*	
C28	−0.4097 (2)	0.6165 (2)	−0.04147 (11)	0.0685 (6)	
H28	−0.4294	0.5342	−0.0392	0.082*	
C29	−0.4949 (2)	0.7035 (3)	−0.05576 (13)	0.0823 (8)	
H29	−0.5726	0.6809	−0.0625	0.099*	
C30	−0.46592 (19)	0.8242 (3)	−0.06011 (13)	0.0828 (8)	
H30	−0.5241	0.8838	−0.0700	0.099*	
C31	−0.35080 (16)	0.85816 (19)	−0.04987 (10)	0.0589 (6)	
H31	−0.3318	0.9404	−0.0533	0.071*	
C32	0.16275 (15)	0.70167 (16)	0.19162 (8)	0.0397 (4)	
C33	0.1692 (2)	0.7890 (3)	0.29487 (10)	0.0889 (9)	
H33A	0.1394	0.7163	0.3103	0.133*	
H33B	0.1411	0.8604	0.3125	0.133*	
H33C	0.2529	0.7878	0.3089	0.133*	
N1	−0.11824 (12)	0.85767 (12)	−0.07964 (7)	0.0383 (4)	
N2	−0.00009 (11)	0.88050 (12)	−0.06145 (6)	0.0348 (3)	
O1	0.16074 (9)	0.85152 (10)	0.03056 (5)	0.0396 (3)	
C16	0.14938 (15)	0.60844 (15)	0.08254 (9)	0.0414 (4)	
H16A	0.1373	0.6280	0.0364	0.050*	
H16B	0.2312	0.5884	0.1005	0.050*	
O3	0.21895 (13)	0.61598 (12)	0.21744 (6)	0.0634 (4)	
O4	0.13024 (12)	0.79288 (12)	0.22348 (6)	0.0590 (4)	

### Atomic displacement parameters (Å^2^)

**Table:**

	*U*^11^	*U*^22^	*U*^33^	*U*^12^	*U*^13^	*U*^23^
C1	0.0438 (10)	0.0316 (9)	0.0343 (9)	0.0017 (7)	0.0112 (7)	0.0032 (7)
C2	0.0536 (12)	0.0533 (11)	0.0370 (10)	−0.0019 (9)	0.0097 (9)	0.0021 (8)
C3	0.0802 (16)	0.0792 (15)	0.0332 (10)	−0.0054 (12)	0.0164 (10)	0.0021 (10)
C4	0.0790 (16)	0.0881 (17)	0.0552 (14)	−0.0065 (13)	0.0346 (12)	0.0133 (12)
C5	0.0556 (13)	0.0908 (17)	0.0597 (14)	−0.0093 (12)	0.0203 (11)	0.0206 (12)
C6	0.0492 (12)	0.0646 (12)	0.0421 (11)	−0.0050 (10)	0.0097 (9)	0.0119 (9)
C7	0.0345 (9)	0.0336 (9)	0.0306 (9)	0.0014 (7)	0.0034 (7)	0.0014 (7)
C8	0.0398 (10)	0.0387 (9)	0.0286 (9)	0.0014 (7)	0.0052 (7)	0.0016 (7)
C9	0.0392 (10)	0.0453 (10)	0.0355 (10)	−0.0040 (8)	0.0030 (8)	0.0087 (8)
C10	0.0423 (11)	0.0698 (13)	0.0540 (12)	−0.0001 (10)	0.0104 (9)	0.0010 (10)
C11	0.0376 (12)	0.0990 (18)	0.0830 (17)	0.0050 (11)	0.0135 (12)	0.0177 (14)
C12	0.0410 (13)	0.133 (2)	0.0752 (18)	−0.0137 (14)	−0.0012 (13)	0.0407 (17)
C13	0.0637 (16)	0.128 (2)	0.0460 (13)	−0.0416 (15)	−0.0112 (12)	0.0112 (14)
C14	0.0566 (13)	0.0784 (14)	0.0430 (11)	−0.0212 (11)	0.0047 (10)	−0.0017 (10)
C15	0.0365 (9)	0.0359 (9)	0.0287 (9)	0.0023 (7)	0.0064 (7)	0.0017 (7)
O2	0.0600 (9)	0.0364 (7)	0.0816 (10)	−0.0005 (6)	0.0295 (8)	−0.0085 (6)
C17	0.0499 (11)	0.0406 (10)	0.0369 (10)	−0.0043 (8)	0.0077 (8)	0.0007 (8)
C18	0.0671 (14)	0.0420 (11)	0.0510 (12)	−0.0099 (10)	0.0078 (10)	−0.0007 (9)
C19	0.0683 (15)	0.0590 (14)	0.0527 (13)	−0.0229 (11)	0.0038 (11)	0.0119 (10)
C20	0.0508 (12)	0.0781 (16)	0.0574 (13)	−0.0135 (11)	0.0160 (10)	0.0157 (11)
C21	0.0474 (12)	0.0582 (12)	0.0511 (12)	−0.0007 (9)	0.0155 (9)	0.0119 (9)
C22	0.0400 (10)	0.0412 (10)	0.0304 (9)	−0.0014 (7)	0.0052 (7)	0.0049 (7)
C23	0.0361 (9)	0.0344 (9)	0.0306 (9)	0.0032 (7)	0.0077 (7)	0.0017 (7)
C24	0.0354 (9)	0.0315 (8)	0.0317 (9)	0.0019 (7)	0.0075 (7)	0.0007 (7)
C25	0.0386 (10)	0.0336 (9)	0.0323 (9)	−0.0001 (7)	0.0067 (7)	−0.0004 (7)
C26	0.0361 (10)	0.0492 (11)	0.0298 (9)	−0.0017 (8)	0.0045 (7)	−0.0003 (7)
C27	0.0477 (12)	0.0523 (11)	0.0494 (12)	−0.0088 (9)	0.0113 (9)	−0.0032 (9)
C28	0.0628 (15)	0.0766 (15)	0.0662 (15)	−0.0275 (12)	0.0177 (12)	−0.0068 (12)
C29	0.0417 (13)	0.124 (2)	0.0783 (17)	−0.0230 (15)	0.0107 (12)	−0.0044 (16)
C30	0.0375 (13)	0.104 (2)	0.099 (2)	0.0145 (13)	0.0047 (12)	0.0103 (16)
C31	0.0408 (12)	0.0642 (13)	0.0651 (14)	0.0062 (9)	0.0032 (10)	0.0074 (10)
C32	0.0392 (10)	0.0408 (10)	0.0370 (10)	0.0023 (8)	0.0066 (8)	0.0043 (8)
C33	0.118 (2)	0.113 (2)	0.0290 (11)	0.0323 (17)	0.0092 (12)	−0.0018 (12)
N1	0.0350 (8)	0.0413 (8)	0.0354 (8)	−0.0010 (6)	0.0044 (6)	0.0021 (6)
N2	0.0343 (8)	0.0374 (8)	0.0305 (7)	−0.0002 (6)	0.0051 (6)	0.0046 (6)
O1	0.0354 (7)	0.0497 (7)	0.0307 (6)	−0.0011 (5)	0.0039 (5)	0.0076 (5)
C16	0.0434 (10)	0.0387 (10)	0.0429 (10)	0.0022 (8)	0.0132 (8)	0.0009 (8)
O3	0.0791 (10)	0.0559 (8)	0.0451 (8)	0.0196 (7)	−0.0004 (7)	0.0125 (6)
O4	0.0785 (10)	0.0661 (9)	0.0288 (7)	0.0220 (7)	0.0082 (6)	−0.0019 (6)

### Geometric parameters (Å, º)

**Table:**

C1—C6	1.375 (2)	C17—C18	1.384 (3)
C1—C2	1.382 (2)	C18—C19	1.364 (3)
C1—N2	1.422 (2)	C18—H18	0.9300
C2—C3	1.377 (3)	C19—C20	1.378 (3)
C2—H2	0.9300	C19—H19	0.9300
C3—C4	1.366 (3)	C20—C21	1.379 (3)
C3—H3	0.9300	C20—H20	0.9300
C4—C5	1.367 (3)	C21—C22	1.384 (3)
C4—H4	0.9300	C21—H21	0.9300
C5—C6	1.374 (3)	C22—C23	1.501 (2)
C5—H5	0.9300	C23—C24	1.513 (2)
C6—H6	0.9300	C23—H23	0.9800
C7—O1	1.3472 (19)	C24—C25	1.420 (2)
C7—N2	1.355 (2)	C25—N1	1.327 (2)
C7—C24	1.356 (2)	C25—C26	1.478 (2)
C8—O1	1.4500 (19)	C26—C27	1.378 (2)
C8—C9	1.496 (2)	C26—C31	1.379 (3)
C8—C15	1.553 (2)	C27—C28	1.372 (3)
C8—H8	0.9800	C27—H27	0.9300
C9—C14	1.377 (3)	C28—C29	1.360 (3)
C9—C10	1.386 (3)	C28—H28	0.9300
C10—C11	1.373 (3)	C29—C30	1.368 (4)
C10—H10	0.9300	C29—H29	0.9300
C11—C12	1.354 (4)	C30—C31	1.380 (3)
C11—H11	0.9300	C30—H30	0.9300
C12—C13	1.363 (4)	C31—H31	0.9300
C12—H12	0.9300	C32—O3	1.190 (2)
C13—C14	1.384 (3)	C32—O4	1.315 (2)
C13—H13	0.9300	C33—O4	1.455 (2)
C14—H14	0.9300	C33—H33A	0.9600
C15—C16	1.517 (2)	C33—H33B	0.9600
C15—C32	1.526 (2)	C33—H33C	0.9600
C15—C23	1.549 (2)	N1—N2	1.3772 (19)
O2—C17	1.355 (2)	C16—H16A	0.9700
O2—C16	1.420 (2)	C16—H16B	0.9700
C17—C22	1.384 (2)		
			
C6—C1—C2	120.15 (16)	C19—C20—C21	119.2 (2)
C6—C1—N2	121.12 (15)	C19—C20—H20	120.4
C2—C1—N2	118.73 (15)	C21—C20—H20	120.4
C3—C2—C1	119.27 (18)	C20—C21—C22	121.58 (19)
C3—C2—H2	120.4	C20—C21—H21	119.2
C1—C2—H2	120.4	C22—C21—H21	119.2
C4—C3—C2	120.86 (19)	C21—C22—C17	118.11 (16)
C4—C3—H3	119.6	C21—C22—C23	122.26 (16)
C2—C3—H3	119.6	C17—C22—C23	119.60 (16)
C3—C4—C5	119.3 (2)	C22—C23—C24	116.12 (13)
C3—C4—H4	120.3	C22—C23—C15	108.25 (13)
C5—C4—H4	120.3	C24—C23—C15	106.87 (13)
C4—C5—C6	121.1 (2)	C22—C23—H23	108.5
C4—C5—H5	119.5	C24—C23—H23	108.5
C6—C5—H5	119.5	C15—C23—H23	108.5
C5—C6—C1	119.30 (18)	C7—C24—C25	103.30 (14)
C5—C6—H6	120.4	C7—C24—C23	120.77 (14)
C1—C6—H6	120.4	C25—C24—C23	135.88 (15)
O1—C7—N2	120.94 (14)	N1—C25—C24	111.68 (14)
O1—C7—C24	128.72 (14)	N1—C25—C26	116.71 (14)
N2—C7—C24	110.29 (14)	C24—C25—C26	131.59 (15)
O1—C8—C9	106.82 (13)	C27—C26—C31	118.50 (17)
O1—C8—C15	109.99 (12)	C27—C26—C25	121.20 (16)
C9—C8—C15	115.48 (13)	C31—C26—C25	120.17 (16)
O1—C8—H8	108.1	C28—C27—C26	120.8 (2)
C9—C8—H8	108.1	C28—C27—H27	119.6
C15—C8—H8	108.1	C26—C27—H27	119.6
C14—C9—C10	118.36 (18)	C29—C28—C27	120.4 (2)
C14—C9—C8	119.10 (17)	C29—C28—H28	119.8
C10—C9—C8	122.54 (16)	C27—C28—H28	119.8
C11—C10—C9	120.9 (2)	C28—C29—C30	119.7 (2)
C11—C10—H10	119.5	C28—C29—H29	120.1
C9—C10—H10	119.5	C30—C29—H29	120.1
C12—C11—C10	120.0 (2)	C29—C30—C31	120.3 (2)
C12—C11—H11	120.0	C29—C30—H30	119.8
C10—C11—H11	120.0	C31—C30—H30	119.8
C11—C12—C13	120.2 (2)	C26—C31—C30	120.3 (2)
C11—C12—H12	119.9	C26—C31—H31	119.9
C13—C12—H12	119.9	C30—C31—H31	119.9
C12—C13—C14	120.5 (2)	O3—C32—O4	124.26 (16)
C12—C13—H13	119.8	O3—C32—C15	124.45 (16)
C14—C13—H13	119.8	O4—C32—C15	111.28 (14)
C9—C14—C13	119.9 (2)	O4—C33—H33A	109.5
C9—C14—H14	120.0	O4—C33—H33B	109.5
C13—C14—H14	120.0	H33A—C33—H33B	109.5
C16—C15—C32	109.52 (13)	O4—C33—H33C	109.5
C16—C15—C23	109.22 (13)	H33A—C33—H33C	109.5
C32—C15—C23	110.59 (13)	H33B—C33—H33C	109.5
C16—C15—C8	110.55 (14)	C25—N1—N2	105.72 (12)
C32—C15—C8	107.64 (13)	C7—N2—N1	109.00 (12)
C23—C15—C8	109.31 (12)	C7—N2—C1	131.03 (14)
C17—O2—C16	119.54 (13)	N1—N2—C1	119.23 (12)
O2—C17—C22	123.89 (15)	C7—O1—C8	111.98 (12)
O2—C17—C18	115.69 (16)	O2—C16—C15	114.68 (14)
C22—C17—C18	120.38 (18)	O2—C16—H16A	108.6
C19—C18—C17	120.47 (19)	C15—C16—H16A	108.6
C19—C18—H18	119.8	O2—C16—H16B	108.6
C17—C18—H18	119.8	C15—C16—H16B	108.6
C18—C19—C20	120.14 (19)	H16A—C16—H16B	107.6
C18—C19—H19	119.9	C32—O4—C33	117.05 (15)
C20—C19—H19	119.9		
			
C6—C1—C2—C3	0.4 (3)	O1—C7—C24—C23	3.7 (3)
N2—C1—C2—C3	−179.34 (17)	N2—C7—C24—C23	−178.75 (13)
C1—C2—C3—C4	0.6 (3)	C22—C23—C24—C7	−141.22 (16)
C2—C3—C4—C5	−1.1 (4)	C15—C23—C24—C7	−20.34 (19)
C3—C4—C5—C6	0.6 (4)	C22—C23—C24—C25	41.6 (3)
C4—C5—C6—C1	0.3 (3)	C15—C23—C24—C25	162.47 (17)
C2—C1—C6—C5	−0.9 (3)	C7—C24—C25—N1	0.66 (18)
N2—C1—C6—C5	178.87 (17)	C23—C24—C25—N1	178.18 (16)
O1—C8—C9—C14	141.20 (16)	C7—C24—C25—C26	179.51 (17)
C15—C8—C9—C14	−96.15 (19)	C23—C24—C25—C26	−3.0 (3)
O1—C8—C9—C10	−39.1 (2)	N1—C25—C26—C27	116.90 (18)
C15—C8—C9—C10	83.5 (2)	C24—C25—C26—C27	−61.9 (2)
C14—C9—C10—C11	1.9 (3)	N1—C25—C26—C31	−58.8 (2)
C8—C9—C10—C11	−177.79 (18)	C24—C25—C26—C31	122.4 (2)
C9—C10—C11—C12	0.7 (3)	C31—C26—C27—C28	0.4 (3)
C10—C11—C12—C13	−2.6 (4)	C25—C26—C27—C28	−175.35 (18)
C11—C12—C13—C14	2.0 (4)	C26—C27—C28—C29	−1.2 (3)
C10—C9—C14—C13	−2.5 (3)	C27—C28—C29—C30	1.0 (4)
C8—C9—C14—C13	177.18 (18)	C28—C29—C30—C31	−0.2 (4)
C12—C13—C14—C9	0.6 (4)	C27—C26—C31—C30	0.4 (3)
O1—C8—C15—C16	54.65 (17)	C25—C26—C31—C30	176.2 (2)
C9—C8—C15—C16	−66.31 (18)	C29—C30—C31—C26	−0.5 (4)
O1—C8—C15—C32	174.23 (12)	C16—C15—C32—O3	7.7 (2)
C9—C8—C15—C32	53.27 (18)	C23—C15—C32—O3	128.08 (18)
O1—C8—C15—C23	−65.61 (16)	C8—C15—C32—O3	−112.56 (19)
C9—C8—C15—C23	173.44 (13)	C16—C15—C32—O4	−173.82 (14)
C16—O2—C17—C22	2.7 (3)	C23—C15—C32—O4	−53.41 (18)
C16—O2—C17—C18	−175.26 (15)	C8—C15—C32—O4	65.95 (18)
O2—C17—C18—C19	177.76 (17)	C24—C25—N1—N2	−0.31 (18)
C22—C17—C18—C19	−0.3 (3)	C26—C25—N1—N2	−179.34 (13)
C17—C18—C19—C20	−1.7 (3)	O1—C7—N2—N1	178.36 (13)
C18—C19—C20—C21	1.6 (3)	C24—C7—N2—N1	0.63 (18)
C19—C20—C21—C22	0.5 (3)	O1—C7—N2—C1	8.5 (3)
C20—C21—C22—C17	−2.4 (3)	C24—C7—N2—C1	−169.21 (15)
C20—C21—C22—C23	179.53 (16)	C25—N1—N2—C7	−0.18 (17)
O2—C17—C22—C21	−175.60 (16)	C25—N1—N2—C1	171.04 (13)
C18—C17—C22—C21	2.3 (3)	C6—C1—N2—C7	−19.0 (3)
O2—C17—C22—C23	2.5 (3)	C2—C1—N2—C7	160.76 (16)
C18—C17—C22—C23	−179.60 (16)	C6—C1—N2—N1	172.05 (15)
C21—C22—C23—C24	−93.1 (2)	C2—C1—N2—N1	−8.2 (2)
C17—C22—C23—C24	88.85 (18)	N2—C7—O1—C8	165.39 (14)
C21—C22—C23—C15	146.77 (16)	C24—C7—O1—C8	−17.3 (2)
C17—C22—C23—C15	−31.3 (2)	C9—C8—O1—C7	172.87 (13)
C16—C15—C23—C22	53.54 (17)	C15—C8—O1—C7	46.85 (17)
C32—C15—C23—C22	−67.05 (16)	C17—O2—C16—C15	23.4 (2)
C8—C15—C23—C22	174.61 (13)	C32—C15—C16—O2	69.47 (18)
C16—C15—C23—C24	−72.22 (16)	C23—C15—C16—O2	−51.78 (18)
C32—C15—C23—C24	167.19 (13)	C8—C15—C16—O2	−172.09 (13)
C8—C15—C23—C24	48.85 (16)	O3—C32—O4—C33	1.9 (3)
O1—C7—C24—C25	−178.27 (15)	C15—C32—O4—C33	−176.62 (18)
N2—C7—C24—C25	−0.76 (17)		

### Hydrogen-bond geometry (Å, º)

*Cg*1, *Cg*4 and *Cg*6 are the centroids of the N1/N2/C7/C24/C25, C1–C6 and C17–C22 rings, respectively.

**Table:**

*D*—H···*A*	*D*—H	H···*A*	*D*···*A*	*D*—H···*A*
C18—H18···N2^i^	0.93	2.62	3.517 (3)	163
C6—H6···O1	0.93	2.26	2.877 (2)	123
C13—H13···*Cg*6^ii^	0.93	2.98	3.904 (8)	174
C18—H18···*Cg*1^i^	0.93	2.88	3.720 (5)	150
C23—H23···*Cg*4^iii^	0.98	2.86	3.787 (6)	159

Symmetry codes: (i) −*x*, −*y*+1, −*z*; (ii) −*x*+1/2, *y*+1/2, −*z*+1/2; (iii) −*x*, −*y*+2, −*z*.

## References

[bb1] Brooks, G. T. (1998). *Pestic. Sci.* **22**, 41–50.

[bb2] Bruker (2008). *APEX2* and *SAINT* Bruker AXS Inc., Madison, Wisconsin, USA.

[bb3] Cai, S. X., Drewe, J. & Kasibhatla, S. (2006). *Curr. Med. Chem.* **13**, 2627–2644. 10.2174/092986706778201521 17017915

[bb4] Gabor, M. (1988). *The Pharmacology of Benzopyrone Derivatives and Related Compounds*, pp. 91–126. Budapest: Akademiai Kiado.

[bb5] Ponnusamy, R., Sabari, V., Sivakumar, G., Bakthadoss, M. & Aravindhan, S. (2013). *Acta Cryst.* E**69**, o267–o268. 10.1107/S1600536813001244 PMC3569797 23424543

[bb6] Sheldrick, G. M. (1996). *SADABS* University of Göttingen, Germany.

[bb7] Sheldrick, G. M. (2008). *Acta Cryst.* A**64**, 112–122. 10.1107/S0108767307043930 18156677

[bb8] Spek, A. L. (2009). *Acta Cryst.* D**65**, 148–155. 10.1107/S090744490804362X PMC2631630 19171970

[bb9] Tang, Q.-G., Wu, W.-Y., He, W., Sun, H.-S. & Guo, C. (2007). *Acta Cryst.* E**63**, o1437–o1438.

[bb10] Valenti, P., Da Re, P., Rampa, A., Montanari, P., Carrara, M. & Cima, L. (1993). *Anticancer Drug. Des.* **8**, 349–360. 8251042

